# Review 2: Primary graft dysfunction after lung transplant—pathophysiology, clinical considerations and therapeutic targets

**DOI:** 10.1007/s00540-020-02823-6

**Published:** 2020-07-20

**Authors:** Zhaosheng Jin, Ka Chun Suen, Zhiping Wang, Daqing Ma

**Affiliations:** 1 Anaesthetics, Pain Medicine and Intensive Care, Department of Surgery and Cancer, Faculty of Medicine, Imperial College London, Chelsea and Westminster Hospital, London, SW10 9NH UK; 2 Department of Anesthesiology, Affiliated Hospital of Xuzhou Medical University, Xuzhou, Jiangsu China

**Keywords:** Lung transplantation, Primary graft dysfunction, Reperfusion injury, Therapeutics

## Abstract

Primary graft dysfunction (PGD) is one of the most common complications in the early postoperative period and is the most common cause of death in the first postoperative month. The underlying pathophysiology is thought to be the ischaemia–reperfusion injury that occurs during the storage and reperfusion of the lung engraftment; this triggers a cascade of pathological changes, which result in pulmonary vascular dysfunction and loss of the normal alveolar architecture. There are a number of surgical and anaesthetic factors which may be related to the development of PGD. To date, although treatment options for PGD are limited, there are several promising experimental therapeutic targets. In this review, we will discuss the pathophysiology, clinical management and potential therapeutic targets of PGD.

## Introduction

Since the first successful lung transplant in 1981, there have been tremendous advances in technology, surgical and anaesthetic techniques, as well as postoperative care of lung transplant patients [1]. The morbidity and mortality rates are considerably lower compared to a decade ago, and it is estimated that more than 4000 lung transplants are now carried out each year worldwide [2, 3]. However, the early postoperative period remains a perilous time for the patients. It is estimated that approximately 7% of lung transplant patients dies within the first month after the operation, and more will suffer complications [2].

Primary graft dysfunction (PGD) is one of the most frequent complications in the early postoperative period. It occurs in approximately 10% of the recipients, and is associated with 42% mortality in the month after transplant, sevenfold increase compared to patients without PGD [4]. In our previous review, we discussed the pre-transplantation issues with donor and graft selection, graft harvesting and storage. In this review, we will discuss the pathophysiology of ischaemia reperfusion injury (IRI) and PGD.

## Primary graft dysfunction

PGD is the most common cause of death in the first 30 days after lung transplant [2]. It occurs usually within the first 3 days after lung transplant as demonstrated by hypoxemia secondary to impaired gas exchange and is associated with non-cardiogenic pulmonary infiltrates on chest radiograph [5]. It can be classified according to the Internal Society of Heart and Lung Transplant (ISHLT) grading system based on the arterial O_2_ partial pressure to fraction of inspired O_2_ (PaO_2_/FiO_2_) ratio and the presence of chest radiograph findings. High-grade graft dysfunction is defined as the presence of both high PaO_2_/FiO_2_ ratio and radiological pulmonary infiltrate which is associated with significantly worse prognosis [6]. It has been found that more severe PGD as defined by the ISHLT grading system is associated with significantly worse prognosis [7]. In most lung transplant cases, the graft vasculature is flushed with preservative solution which is later kept in cold storage for the transfer process. It has been noted in animal studies that even without the transplant surgery, iatrogenic lung ischaemia and subsequent reperfusion results in respiratory and haemodynamic changes similar to that of PGD, demonstrating hypoxia, pulmonary oedema and reduced lung compliance [8–10]. It is, therefore, thought that IRI is likely to play a significant role in the development of PGD [11].

## Ischaemia reperfusion injury

### Energy depletion

As discussed in our previous review, ischaemia can very rapidly lead to a cascade of pathological changes in the cells. High-energy phosphate becomes depleted, which impairs a wide range of vital cellular functions. The lack of Na^+^/K^+^ ATPase action results in rapid loss of electrochemical gradients, while influx of sodium causes cell swelling. Dysregulated calcium homeostasis results in calcium accumulation in the cytosol which results in aberrant activation of various signalling systems such as membrane phospholipase and phosphokinases [12, 13]. Ultimately, damage caused by ischaemia results in cell death and the release of damage-associated molecular patterns molecules such as HMGB-1 and HSP-60 [14, 15]. These molecules can bind to receptors such as Toll-like receptors and RAGE, and results in activation of MAPK and nuclear factor kappa-light-chain-enhancer of activated B cells (NF-kB). This eventually leads to the upregulation in cytokine production, which not only activates the alveolar resident macrophages, but also serves as chemo-attractants and activators to circulating immune cells upon reperfusion [16].

During ischaemia, metabolism of ATP results in the accumulation of hypoxanthine, which upon reperfusion induces oxidative stress [11]. In addition, it is thought that lack of shear stress on the vascular endothelium results in upregulated expression of nicotinamide adenine dinucleotide phosphate oxidase (NADPH oxidase) [17]. Subsequently during reperfusion, the upregulated activities of NADPH oxidase results in a surge of reactive oxygen species (ROS) generation.

It has also been demonstrated that ischaemia can result in significant vascular dysfunction. Studies have shown that cold ischaemia which occurs during storage can results in significant vascular endothelial structural changes [18], increasing the vascular permeability [19] while impairing pulmonary vasodilatation [20]. The vascular dysfunction further worsens the pathological changes upon reperfusion.

What is unique in lung graft, however, is its gas exchange surface and ability to maintain oxygenation in ischemic condition. In animal models, preservation of lung graft inflated with 21% oxygen maintained its ATP and lactate level for more than 60 min during warm ischaemia; this is further augmented in grafts inflated with 100% oxygen, which maintained ATP production for almost 5 hours [21]. As such, it is recommended that lung grafts should be stored inflated or ventilated [6].

### Oxidative and nitrosative stress

IRI is often characterised by rapid accumulation of ROS soon after reperfusion, with increased activities of ROS-generating enzymes such as NADPH oxidase, xanthine oxidase and myeloperoxidase [8, 17, 22, 23]. Reduction in anti-oxidative capacity [15, 24] and the occurrence of oxidative stress [24–27] are also observed. NADPH oxidase activity seems to play a significant role in the pathophysiology of IRI, as NADPH oxidase knockout animal demonstrates significantly blunted oxidative stress and inflammatory response to IRI. The lung grafts also displayed improved compliance, reduced pulmonary hypertension and oedema. Similar findings were also seen with NADPH oxidase inhibition [15, 22]. It is thought that NADPH oxidase activity may also enhance the release of chemokines such as (C-X-C motif) ligand 1 (CXCL1), CXCL2, (C–C motif) ligand 2 (CCL 2) and CCL 5 responsible for further recruiting immune cells such as neutrophils to the site of injury [15, 22]. The recruitment of neutrophil leads to a number of detrimental effects on the lung tissue, including further generation of ROS [28].

Another important source of reactive oxygen species is the activity of xanthine oxidase. Xanthine oxidase is an oxidoreductase which catalyses the reaction of hypoxanthine to xanthine, then to uric acid. Both steps generate hydrogen peroxide. As mentioned previously, hypoxia leads to the accumulation of hypoxanthine from the breakdown of ATP [11]. Studies in the past have also suggested that xanthine oxidase conversion from its precursor enzyme xanthine dehydrogenase is augmented by hypoxia and by neutrophil activity [29–31]. The upregulated xanthine oxidase activity, as well as an abundance of substrate leads to the excessive production of ROS. Indeed, it has been demonstrated that xanthine oxidase inhibition in animal models of IRI can significantly reduce the extent of the endothelial dysfunction [32].

In addition, oxidative stress generated by NADPH oxidase may also have an impact on the nitric oxide synthase (NOS) function. Oxidation of the NOS co-factor tetrahydrobiopterin uncouples NOS function, and causes it to generate ROS instead of nitric oxide, which further adds to the oxidative burden [33, 34]. In the presence of excessive ROS, nitric oxide may also be converted to peroxynitrite, a reactive nitrogen species which could cause oxidation and nitration injury [35].

### Inflammatory response

IRI is associated with a cascade of proinflammatory changes, including the upregulation of cytokines such as tumour necrosis factor-α (TNF-α), Interleukin-1β (IL-1β), IL-6; chemokines such as CCL1, CCL5, CXCL2 (IL-8); as well as immune cell adhesion molecules such as integrin, intercellular adhesion molecule 1 (ICAM 1) and vascular cell adhesion protein (VCAM) [36–38]. It is thought that TNF-α upregulates NF-κB by inhibiting IκB, which further upregulates TNF-α production and other inflammatory mediators as mentioned above [15, 36, 38, 39]. This promotes the chemotaxis of inflammatory cells such as neutrophils and natural killer cells.

Indeed, IRI is characterised by neutrophil infiltration and neutrophil activity, and inhibiting neutrophil function reduces the extent of ischaemia reperfusion related damage [23, 24, 38, 40], while IL-17, produced by natural killer T cell, is another potent neutrophil chemoattractant [41].

### Outcome of injury: vascular dysfunction and loss of alveolar architecture

Pre-clinical studies showed that lung IRI is characteristically associated with pulmonary hypertension and increase in the lung water content. Cold storage could impair the function of both the endothelial barrier and the vascular smooth muscles [18–20]. In addition, both the recruited neutrophil and ROS generation during the reperfusion has also been shown to cause increase in permeability [22, 42–44]. The injury has also been associated with impaired vascular smooth muscle relaxation, leading to pulmonary hypertension [45, 46]. It has been suggested that this is likely mediated by HIF-1α, iNOS and ATP dependent potassium channels [47, 48].

The combination of cell death, inflammatory changes and pulmonary vascular dysfunction leads to widespread changes to the microscopic architecture of the alveolar space, with interstitial oedema, neutrophil infiltration, hyaline membrane formation and intra-alveolar haemorrhages [49–51]. Not surprisingly, this impairs both the alveolar gas exchange as well as the ventilation mechanics.

### Pre-transplant risk factors of PGD

Clinically, both donor and recipient factors could impact on the occurrence of PGD. Donor factors include age and history of smoking, gender and pre-existing illness [52–55]. Recipient factors include BMI, gender, pre-existing lung pathologies such as sarcoidosis, pulmonary fibrosis and pulmonary hypertension [52, 56, 57]. Prolonged ischaemic time is also associated with significantly higher risk of PGD [52].

It is estimated that PGD accounts for almost 1 in 4 post-transplant deaths in the first month [55, 58]. In recent years, extensive research has been done in attempt to reduce the development of PGD, including shortening ischaemic time, controlled reperfusion, and various medications and IRI pathway modulations [59–62]; we will discuss this in detail in the next section.

## Surgical and anaesthetic consideration in PGD prevention

### Reperfusion considerations

As described above, repurfusion and ventilation of lung graft are closely linked to the development of IRI and subsequent PGD. Introduction of oxygen to the ischaemic lung graft could result in worsened oxidative stress. The pulmonary vasculature is often constricted which results in pulmonary hypertension, and reperfusion induces a myriad of immune cells which worsens inflammation. This would make the reperfusion process a therapeutic target of preventing PGD [57, 63]. Due to pulmonary vascular constriction, a sudden restoration of circulation can result in sharp increase in the pulmonary artery pressure. A few studies have looked into the effect of varying rates of reperfusion on the function of the lung graft, and found that initial perfusion the lung graft at a lower flow rate is associated with significantly less pulmonary oedema and reduced shunting, which results in improved lung compliance and gas exchange [64–67].

Indeed, Ardehal et al. conducted a clinical study where the lung graft was perfused with of a modified perfusate via pressure-controlled delivery for 10 min, before pulmonary blood flow was re-established. They reported that the modified perfusion was associated with a significantly lower rate of PGD [63].

In addition to the pressure-controlled delivery, Ardehal also reported a number of modifications to the perfusate, this includes leucodepletion [63]. There are also animal studies which suggest that leucodepletion during both ex vivo lung perfusion (EVLP) and reperfusion after engraftment results in improved graft function [68, 69].

Diamond et al. conducted a multicentre retrospective study of 1255 lung transplant patients, and found that the only significant perioperative risk factors associated with PGD were FiO_2_ during reperfusion, single lung transplant and cardiopulmonary bypass. The study went on further to illustrate that the odd ratio for PGD increases by 10% for every 10% increase in FiO_2_ at the time of reperfusion [57]. While the authors acknowledged that high oxygen requirement is an indication of poor graft function, they also commented that the observed variation of reperfusion FiO_2_ may be partly due to clinician preference and suggested judicious FiO_2_ setting may be a modifiable risk factor in PGD. Studies directly comparing liberal vs restrictive oxygen therapy at the time of reperfusion are warranted.

### Inhaled nitric oxide

Nitric oxide is known to possess vasodilatory effect to blood vessels by relaxing smooth muscle cells through increasing cGMP. It was thought that inhaled nitric oxide (iNO) may bring localised, vasodilatory effect to pulmonary vasculature, improving oxygenation [70, 71]. Clinical studies have showed the use of iNO in acute respiratory distress syndrome (ARDS) is associated with improved oxygenation, although no impact on mortality is observed [72]. Some evidence showed that iNO may be beneficial on the prevention and treatment of PGD in lung transplantation. A small study that included 6 patients has shown that the administration of iNO immediately after lung transplant reduces pulmonary artery pressure and improves oxygenation [73] Another study investigated the effect of postoperative administration of iNO for a period of 84 h on average, on those who had already developed PGD. It showed that iNO improves the arterial oxygenation and reduces pulmonary artery pressure [70]. In contrast, a randomised controlled trial showed that iNO may not be beneficial in treatment of PGD [74]. In one study, iNO or placebo were administered 10 min after the reperfusion at lung transplantation. It showed that there is no difference on PaO_2_/FiO_2_ ratio, successful extubation and intensive care unit discharge time or 30-day postoperative mortality [74]. Studies also focused on the preventative role of iNO on PGD. Evidence has shown that prophylactic iNO does not prevent the development of PGD but may improve oxygenation in those who have already developed PGD [75]. A similar result was also seen in a study that looked in the effect of prophylactic use of iNO during the first 30 min of reperfusion showed no impact on preventing PGD [76] Another randomised controlled trial study also showed that prophylactic iNO has no impact on the development of pulmonary oedema [77]. iNO exposure could theoretically lead to the formation of methaemoglobin (MetHb), but the risk of significant methaemoglobinaemia is minimal at the therapeutic doses. A Cochrane review of 679 patients who received iNO therapy reported 4 cases where MetHb were above 5% [72]. On the other hand, the risk of iNO induced methaemoglobinaemia in children may be significant [78]. In addition, there have been cases of renal failure observed with iNO exposure [70, 72].

### Inhaled prostaglandin

Prostaglandin (PGE1) can cause vasodilation by increasing the intracellular cyclic adenosine monophosphate (cAMP) [79]. Similar to iNO, it was thought that inhaled PGE1 may bring vasodilatory effect to the lungs and improve oxygenation [80]. PGE1 may also reduce the expression of proinflammatory mediators such as TNF-α, IL12; and promote expression of IL 10 after IRI [81]. Other studies have reported that PGE1 reduces the expression of vascular adhesion molecules and monocyte recruitment, while increasing the expression of IL 6 [82, 83]. A meta-analysis of 25 studies has suggested that inhaled PGE1 may improve oxygenation in patients with ARDS [84]. A study evaluated the effect of intraoperative administration of aerosol PGE1 during lung transplantation. Compared to the patient’s baseline, an improvement on PaO_2_/FiO_2_ ratio and reduction in pulmonary arterial pressure are observed, whilst the haemodynamics remain unchanged [85]. Another study evaluated the effect of donor PGE1 bolus before cross clamping plus PGE1 addition to the graft preservation fluid, and reported that the intervention is associated with significantly increased long-term survival (odds ratio = 9.8) [86]. It has also been suggested that PGE1 infusion may be beneficial as a rescue treatment in patients with severe PGD [87].

### Positive end expiratory pressure (PEEP)

The use of PEEP as a ventilation strategy has been shown to improve pulmonary gas exchange and oxygenation, decrease work of breathing [88, 89]. Evidence showed that high PEEP may reduce mortality in patients with ARDS [90]. Animal and clinical studies have shown that higher PEEP may also play a role in prevention of PGD after lung transplant. A study was carried out to compare higher and lower PEEP on unilateral lung transplant on pigs. It showed that higher PEEP (10 cmH_2_O) compared to lower PEEP (5 cmH_2_O) has an impact on increased compliance and reduced airway resistance, although no difference is observed on PaO_2_ level [91]. A randomised controlled study investigated the effect of open lung protective ventilation on lung transplant [92]. They compared the control group using 5 cmH_2_O PEEP and tidal volume of 6 ml kg^−1^ for 2 lung ventilation and 4 ml kg^−1^ for 1 lung ventilation and the study group using pressure-controlled ventilation of 16 cmH_2_O with 10 cmH_2_O PEEP and recruitment manoeuvres [92]. It was shown that the group with higher PEEP and the use of recruitment manoeuvres is associated with an improvement of PaO_2_/FiO_2_ as well as shorter time for tracheal extubation. However, a prolonged effect on the improvement of PaO_2_/FiO_2_was not observed.

### β-Adrenoceptor agonists

β_2_-Adrenoceptors are distributed throughout the lung tissue. β_2_-Adrenoceptors agonists are commonly used for its bronchodilating effect; however, other proposed effects of β_2_-adrenoceptors agonists include the relaxation of vascular smooth muscle, maintenance of the endothelial barrier, and may promote the uptake of alveolar fluid. A number of animal studies have demonstrated that nebulisation of β_2_ selective agonists during ventilated EVLP is associated with significantly lower graft vascular resistance, reduced oedema development, better pulmonary compliance and better gas exchange [23, 93–95]. Similar findings have also been observed in animal recipients of the transplant studies [96, 97]. In addition, a study by Sapru et al. [98] found that single-nucleotide polymorphisms (SNP) in the donor β adrenoceptor genes are associated with significant variations in the graft viability, with higher viability in SNPs with higher isoprenaline sensitivity [99, 100].

To date, the only human study using β_2_ agonist was reported by Ware et al. [73]. They demonstrated that in brain dead critical care patients awaiting transplant work-up, intermittent nebulisation of salbutamol did not result in significant change in the oxygenation or lung compliance. Not surprisingly, significantly more patients in the intervention group developed tachycardia which warranted stopping of the medication [94]. However, it is possible that the effective window of β_2_ agonists is limited to the graft storage period; and that higher dose of β_2_ agonists may be administered to the isolated lung without the cardiovascular side effects [96, 97]. More human studies are needed to clarify if aggressive β_2_ agonist treatments are clinically useful during graft retrieval and implantation process.

### Other interventions

Transfusion-related acute lung injury (TRALI) is defined as acute lung injury within 6 h after a blood transfusion, and is characterised by radiological pulmonary infiltrate and hypoxia. The risk of TRALI is increased by both intrinsic lung injury (as with surgical manipulation of the graft) and systemic insult (as with cardiopulmonary bypass), thus making lung transplant patients high risk for developing TRALI [101]. In addition, there is a possibility that the antibody from the transfused blood may cross react with the lung graft [102]. Judicious use of blood products, as well as close monitoring is required to minimize the risk.

Non-invasive ventilation (NIV) may also be beneficial in patients who have developed PGD [103]. A study investigated the role of NIV in those who had developed acute respiratory failure after lung transplant. After the administration of NIV for an average of 5 days, improved PaO_2_/FiO_2_ratio and reduction in PaCO_2_ were observed [103].

Prone positioning may also be beneficial in protection against PGD. It is possible that ventilating through prone position with the use of non-invasive high-frequency percussion ventilation postoperatively may help with improved gas exchange and mucus clearance [104].

Low-flow venovenous removal of CO_2_ is a method that removes 20–25% of carbon dioxide from blood. A small study including 3 patients was carried out to investigate the beneficial role of venovenous removal of CO_2_. It showed that together with the use of iNO and prostacyclin, a reduction in PaCO_2_, pulmonary infiltrates and increased PaO_2_/FiO_2_ ratio were observed [105]. Similar results on improved PaO_2_/FiO_2_ ratio and pH level were also observed in another study, although one patient eventually died [106].

## Novel therapies for ischaemia–reperfusion injury

**Fig. 1:**
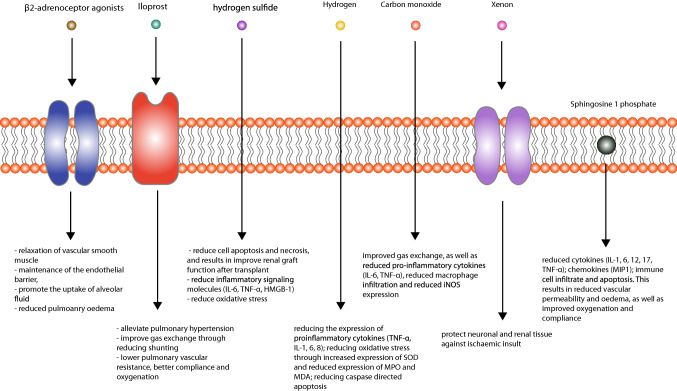
Potential therapeutic agents for primary graft dysfunction and their mechanisms of action

In the last decade, a number of experimental approaches for minimising IRI have been studied and reported in literature, with varying degrees of success. A summary of the therapies and their effects are summarised in Fig. 1.

### Ischaemic pre-conditioning and post-conditioning

Ischaemic pre-conditioning is a process of transient (seconds to minutes) disruption of blood supply, which is thought to have protective effects against further ischaemia and IRI. It can be carried out directly on the tissue of interest, or carried out remotely on another part of the organism [107, 108]. Ischaemic post-conditioning on the other hand is a process of deliberate, cyclic disruption of blood supply after the initial insult [109], which ideally needs to occur within minutes of the initial insult [110].

In animal models of IRI, both direct and remote ischaemic pre-conditionings, as well as ischaemic post-conditioning have been demonstrated to reduce the extent of the IR injury [109, 111, 112]. Ischaemic conditioning has a myriad of effects on the inflammatory response to IR, it reduces the expression of inflammatory cytokines such as TNF-α, IL1b, IL6, CCL2; reduces leucocyte recruitment and extravasation by downregulating ICAM [107, 111, 113]. The reduction in neutrophil chemotaxis and activity also reduces of the production of ROS, as seen by a reduction in MDA and lipid hydroperoxides (LPO) production [27]. In addition, ischaemic conditioning also reduces mediators of apoptosis, including BAX, Caspase, cytochrome C and Fas ligand [108, 112].

Indeed, ischaemic pre-conditioning and post-condition have both demonstrated protective effects against signs of PGD in animal models of lung transplant. In a rat model of lung transplant, Hu et al. reported significantly less pulmonary oedema and better gas exchange in animals underwent ischaemic post-conditioning, with 5 cycles if 1 min occlusion of the pulmonary artery [107]. While Jiang et al. reported significantly less pulmonary oedema and microscopic changes after transplant in animals underwent both direct and remote ischaemic pre-conditioning [49].

In 2014, Lin et al. reported a clinical trial of 60 patients who underwent bilateral lung transplant. Patients were randomised to receive either 3 cycles of lower limb ischaemic pre-conditioning prior to the reperfusion, or the control group. They reported a tendency towards higher alveolar–arterial O_2_ gradient and lower rate of PGD; however, neither were statistically significant [114]. The exact role of ischaemic conditioning on the outcome of lung transplant will need to be explicated through larger clinical trials.

### Prostacyclin analogue

Iloprost is a prostacyclin analogue and a cyclic adenosine monophosphate (cAMP)-mediated pulmonary vasodilator. It is thought that in the context of lung transplant, the pulmonary vasodilatory effect could alleviate pulmonary hypertension and improve gas exchange through reducing shunting [115]. In addition, it is thought to maintain endothelial integrity, and reduce platelet aggregation [116]. Animal studies have shown that when nebulised through the ventilator, or added to the preservative fluid, iloprost administration is associated with better function of the lung graft in the recipient animal, with significantly lower pulmonary vascular resistance, better compliance and oxygenation [45, 46, 117].

In addition, Lee et al. reported that in a cohort of 60 patients, those given nebulised iloprost immediately after reperfusion had significantly lower oxygen requirement and less pulmonary infiltrate, as well as higher compliance [61]. The current literature suggests that iloprost is a promising lung graft protecting agent, and large clinical trials are needed to confirm its benefits.

### Hydrogen sulfide donors

Hydrogen sulfide is a gas with a characteristically pungent odour and is toxic at a high concentration [118]. At a lower concentration, however, it has been shown to have anti-inflammatory, anti-oxidative and anti-apoptotic effects [119, 120].

In animal studies, hydrogen sulfide administered through the ventilator, as well as hydrogen sulfide donor sodium hydrosulfide given systemically to the graft recipients have been shown to reduce the expression of pro-inflammatory cytokines, alleviates oxidative stress, and reduce caspase driven apoptosis. This is associated with an increased pulmonary compliance, increased alveolar gas exchange, and reduction in shunting and oedema [121–124].

### Other inhaled gases

Hydrogen gas has been proposed to be a potent free radical scavenger as well as anti-inflammatory agent [125]. In animal models, insufflating the lung graft with 2–3% hydrogen is associated with significantly better compliance and gas exchange, reduced pulmonary vascular resistance and histological changes. This is thought to be mediated through a number of mechanisms, such as reducing the expression of proinflammatory cytokines (TNF-α, IL-1, 6, 8), reducing oxidative stress and reducing caspase-mediated apoptosis [24, 50, 126].

Although the mechanism is not clear, carbon monoxide is thought to have potent anti-inflammatory properties [127]. In animal models of lung transplant, insufflating the lung graft with 500PPM carbon monoxide was associated with reduced pro-inflammatory mediators, oxidative stress, and apoptosis mediators. In addition, the transplanted lung grafts demonstrated reduced edema and improved compliance and gas exchange [8, 126].

### α1-Antitrypsin

α_1_-Antitrypsin is a protease inhibitor that protects tissue from the proteolytic enzymes produced by inflammatory cells. In addition to the direct inhibitory effect on proteases, it is also thought to have anti-apoptotic properties, regulate macrophage and neutrophil actions, and has been shown to protect against ischaemia/reperfusion injuries in a number of animal models [128].

In animal models of lung transplant, α_1_-antitrypsin administration has been associated with significantly reduced NF-κB expression and neutrophil infiltration, better gas exchange, and better lung compliance [129, 130]. In addition, in EVLP only and in ischaemia/reperfusion models, α_1_-antitrypsin administration has also been associated with significantly reduced inflammatory cytokine expression, neutrophil infiltration and apoptosis, with better lung compliance [62, 131].

### Other novel targets

Sphingosine 1 phosphate (S1P) is a lipid growth factor derived from cell membrane sphingolipids. It has been proposed to promote cell survival, proliferation and angiogenesis [132, 133]. In animal lung transplant models, S1P administration is associated with significantly reduced cytokines, immune cell infiltrate and apoptosis. This results in reduced vascular permeability and oedema, as well as improved oxygenation and compliance [134–136].

Adenosine receptor agonism has been shown to reduce IRI by through its regulatory function on T cells and NK cells [137, 138]. Animal models of IRI have reported that adenosine receptor agonists significantly reduce inflammatory mediator expression, as well as improve lung function [139–143].

Del Sorbo et al. suggested that siRNA against Fas receptor, responsible for apoptosis in ischaemia/reperfusion could reduce graft dysfunction [144].

Necroptosis as a result of the calpain-STAT3-RIPK pathway activation has also been implicated in ischaemia/reperfusion pathology, and it has been suggested that blocking the said pathway may also reduce graft dysfunction [145, 146].

IL-10 is a regulatory cytokine that can inhibit NF-κB and the JAK/STAT pathways. Upregulation of IL-10 has been implicated in the cytoprotective effect of α_1_-antitrypsin [147], and in animal model of lung transplant, IL-10 gene therapy was associated with significantly better gas exchange [51].

## Conclusion

PGD is a common and potentially life-threatening complication which is thought to be secondary to IRI following engraftment. While there are now clear guidelines on its diagnosis, there are limited preventative and treatment options available. We have discussed in detail the pathophysiology of PGD and highlighted some of the therapeutic options reported in pre-clinical studies, which includes some drugs which are already licensed for clinical use. With the expansion of the pre-clinical evidence, it may be possible for some of those experimental therapies to be developed for clinical use.
